# Subependymal giant cell astrocytoma (SEGA): a case report and review of the literature

**DOI:** 10.1186/s13256-016-0818-6

**Published:** 2016-02-09

**Authors:** Layla Tahiri Elousrouti, Meryem Lamchahab, Nawal Bougtoub, Hinde Elfatemi, Laila Chbani, Taoufik Harmouch, Mustapha Maaroufi, Afaf Amarti Riffi

**Affiliations:** 1 Department of Pathology, Hassan II University Hospital, Route Sidi Harazem, 30000 Fes, Morocco; 2 Department of Radiology, Hassan II University Hospital, Route de Sidi, Harazem, 30000 Fes, Morocco

**Keywords:** Histologic analysis, Subependymal giant cell astrocytoma, Tuberous sclerosis complex

## Abstract

**Background:**

Subependymal giant cell astrocytoma is a rare tumor that occurs in the wall of the lateral ventricle and foramen of Monro and, rarely, in the third ventricle. It is one of the intracranial lesions found in tuberous sclerosis complex (which include subependymal nodules, cortical tubers, retinal astrocytoma and subependymal giant cell astrocytoma), but cases without such lesions have also been reported in the literature. It was described for the first time in 1908 by Vogt as part of the typical triad of tuberous sclerosis complex. At the 2012 Washington Consensus Conference, it was decided by the invited expert panel to document the definition of subependymal giant cell astrocytoma as a lesion at the caudothalamic groove with either a size of more than 1 cm in any direction or a subependymal lesion at any location that has shown serial growth on consecutive imaging regardless of size. Most subependymal giant cell astrocytomas will show avid enhancement after contrast administration; however, a growing subependymal lesion even in the absence of enhancement should be considered a subependymal giant cell astrocytoma.

**Case presentation:**

We report a case of subependymal giant cell astrocytoma in a 10-year-old white girl, who had no clinical symptoms of tuberous sclerosis. A computed tomography scan revealed a voluminous mass in her perilateral ventricle. An extemporaneous examination was in favor of a benign ganglioglioma tumor. After fixation in 10 % neutral-buffered formalin, embedding in paraffin and staining with hematoxylin, eosin and safran, the definitive diagnosis was subependymal giant cell astrocytoma.

**Conclusions:**

Subependymal giant cell astrocytoma is a rare tumor of the central nervous system whose diagnosis is based on clinical, radiological, histological and immunohistochemical arguments. For its rarity, we must consider this diagnosis when faced with a mass near the foramen of Monro in the pediatric population even if there are no other features of tuberous sclerosis complex.

## Background

Subependymal giant cell astrocytoma (SEGA) is a rare tumor that occurs the wall of the lateral ventricle and foramen of Monro, and, rarely, in the third ventricle. It is one of the intracranial lesions found in tuberous sclerosis complex (TSC); this lesion is included in the 2012 International Tuberous Sclerosis Complex Consensus Group as a major feature (which includes subependymal nodules, cortical tubers, retinal astrocytoma and SEGA), but cases without such lesions have also been reported in the literature. The histogenesis of this tumor is poorly understood. Previous studies have reported glial (astrocytic or, rarely, ependymal), neuronal or mixed glial-neuronal differentiation. We report a case of subependymal giant cell astrocytoma in a child without clinical symptoms of tuberous sclerosis. We describe the histological and immunohistochemical features of this rare entity.

## Case presentation

**Fig. 1:**
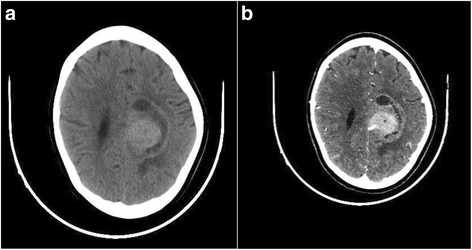
Computed tomography scan revealing a voluminous mass in the perilateral ventricle with similar attenuation to that of cortical gray matter (**a**), following administration of a contrast product, the mass showed marked enhancement (**b**)

**Fig. 2:**
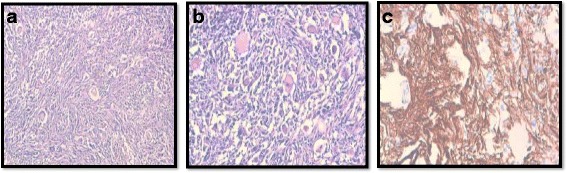
Tumor composed of spindle cells and globular large cells, producing an aspect of ganglion cells: (**a**) hematoxylin-eosin-safran × 100, (**b**) hematoxylin-eosin-safran × 20, (**c**) immunostaining: glial fibrillary acidic protein positive

**Fig. 3:**
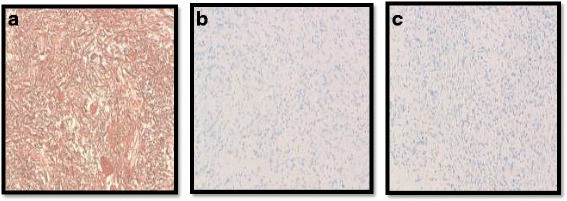
Tumor composed of spindle cells and globular large cells, producing an aspect of ganglion cells: (**a**) immunostaining: PS100 positive (**b**) immunostaining: synaptophysin negative, (**c**) immunostaining: KI67 0 %

A 10-year-old white girl, presented with a 10-month history of convulsive seizures without signs of intracranial hypertension. A computed tomography (CT) scan revealed a voluminous mass in her perilateral ventricle with similar attenuation to that of cortical gray matter (Fig. 1a), following administration of a contrast product, the mass showed marked enhancement (Fig. 1b). Surgery was performed with complete resection of the tumor. An extemporaneous examination was in favor of a benign ganglioglioma tumor. After fixation in 10 % neutral-buffered formalin, embedding in paraffin and staining with hematoxylin, eosin and safran, histologically the tumor was found to be composed of fibrillated spindle cells and globular large cells, with abundant eosinophilic cytoplasm, and voluminous, eccentric nucleus, and large nucleoli, producing an aspect of ganglion cells; mitosis, necrosis, and microvascular proliferation were not rated (Figs. 2a and 2b). Calcifications and perivascular lymphocytes were observed. In immunohistochemical studies, the spindle cells were positive for glial fibrillary acidic protein (GFAP) (Fig. 2c) and S-100 protein (Fig. 3a). Neurofilament and synaptophysin were negative in either the spindle cells or the large cells (Fig. 3b), and KI67 was not detectable (Fig 3c). An examination that included a dermatological evaluation, a retinal examination, and body imaging revealed no stigmata of tuberous sclerosis. The postoperative course was straightforward.

## Discussion

Subependymal giant cell astrocytoma (SEGA) is a slowly growing tumor of unknown histogenesis mainly arising in the periventricular regions adjacent to the foramen of Monro [1, 2], which causes increased intracranial pressure, seizures, and focal neurologic signs. The incidence of SEGAs in tuberous sclerosis (TSC) varies from 5 % to 14 %, and may also be detected prenatally or at birth, although they are much more likely to arise during childhood or adolescence and it would be unusual for one to occur after the age of 20 years if not previously present [1, 3]. At the 2012 Washington Consensus Conference, it was decided by the invited expert panel to document the definition of SEGAs as a lesion at the caudothalamic groove with either a size of more than 1 cm in any direction or a subependymal lesion at any location that has shown serial growth on consecutive imaging regardless of size. Most SEGAs will show avid enhancement after contrast administration; however, a growing subependymal lesion even in the absence of enhancement should be considered a SEGA [4, 2]. Although histologically confirmed SEGA is considered pathognomonic for TSC, there are reports of patients with this tumor who have none of the other stigmata of tuberous sclerosis complex [3, 5]. This is an autosomal dominant phacomatosis, due, in 60 % of cases, to spontaneous mutation in two tumor suppressor genes (TSC1 and TSC2) [6–8]. It is manifested by the development of benign tumors in many organs (heart, kidney, skin, and brain). The cutaneous and neurological involvement is almost constant. The central nervous system is represented by cortical tubers, subependymal nodules, subependymal giant cell astrocytomas and retinal astrocytoma. The risk of mental retardation is high in this condition especially when associated with seizures in the first year of life [9–11]. It typically affects patients during childhood and adolescence; neonatal cases have also been reported [11].

The clinical features of SEGA are due to hydrocephalus, raised intracranial pressure and seizures. Hydrocephalus occurs due to obstruction of the cerebrospinal fluid pathway by the tumor itself [11, 12]. As the association of SEGA and tuberous sclerosis is common, the characteristic symptoms of this disease are often present and must be investigated. In some cases, the SEGA can usher in the absence of clinical stigmata of tuberous sclerosis [7, 10, 13].

CT and magnetic resonance (MR) imaging characteristics are usually nonspecific, and the location of the mass and the patient’s age are useful indicators of the specific tissue diagnosis [9, 13, 14]. Although nonspecific, the CT and MR findings objectify a well-circumscribed mass at the foramen of Monro, which frequently exhibits partial calcification or cyst formation. Enhancement following contrast administration is strong but inhomogeneous [9, 15].

Histologically, subependymal giant cell astrocytomas are characterized by triple cell components distributed over a fibrillar background; large astrocyte-like cells with perivascular pseudopalisading and often calcification. There is a wide spectrum of cells within the tumor cells, ranging from gemistocytic astrocytes to long fibrillated and spindle cells, as well as large giant cells, some of them with a ganglionic appearance with large eosinophilic and finely granular cytoplasm, the nucleus are rounded or oval, large, and eccentric. Increased mitotic activity, pleomorphism, occasional endothelial proliferation, and necrosis did not have any prognostic value [16, 7], perivascular lymphocytic inflammatory infiltrate is usually found, as noted in our case [12, 17].

Immunohistochemical studies have demonstrated a mixed glial and neuronal differentiation of subependymal giant cell astrocytoma [13–19], leading some to hypothesize that these lesions arise from multipotent progenitor cells within the germinal mantle of the developing brain [11, 13]. In our case, only astroglial differentiation was demonstrated by detectable levels of GFAP and S100 protein immunoreactivity and absence of both synaptophysin and neurofilament immunoreactivity. These findings are in agreement with literature data, which demonstrated that astroglial differentiation predominantly occurs in cases of SEGA in asymptomatic tuberous sclerosis, in contrast to both glial and neuronal differentiation in tuberous sclerosis-associated cases [11, 17, 19].

Radical and early surgery is the treatment of choice, it is associated with a better prognosis without complications, due partly to the intracranial hypertension and the association with tuberous sclerosis and the surgical procedure itself, which influences the functional and vital prognosis [5, 20, 21]. Recurrences are related to incomplete surgery.

The progress of targeted therapies has a new perspective in SEGA’s treatment; it is the inhibitor of mTOR (everolimus) that indicates from the age of 12 months in subependymal giant cell astrocytoma associated with tuberous sclerosis complex requiring therapeutic intervention but not eligible for surgical resection. This molecule can reduce the volume of the tumor without destroying it entirely. Furthermore, discontinuation of the drug led to a resumption of tumor growth [22].

## Conclusions

Subependymal giant cell astrocytoma is a rare tumor of the central nervous system whose diagnosis is based on clinical, radiological, histological and immunohistochemical arguments. It should be included in the differential diagnosis of a mass near the foramen of Monro even if there are no other features of tuberous sclerosis complex.

## Consent

Written informed consent was obtained from the patient’s legal guardian(s) for publication of this case report and any accompanying images. A copy of the written consent is available for review by the Editor-in-Chief of this journal.
